# Risk assessment and decision-making for patients undergoing orthopedic surgery

**DOI:** 10.1186/s13018-015-0308-3

**Published:** 2015-10-29

**Authors:** De-ming Bao, Ning Li, Lei Xia

**Affiliations:** Department of Orthopedics, The First Affiliated Hospital of Zhengzhou University, 1 Jianshe Road, Zhengzhou, 450052 China

**Keywords:** Morbidity, Postoperative risk, Ejection fraction

## Abstract

**Purpose:**

Physical and operative severity score for the enumeration of mortality and morbidity (POSSUM) scoring system was designed to predict the postoperative morbidity and mortality mainly in general surgery. The purpose of this study was to assess the value of POSSUM scoring system in predicting outcomes of patients undergoing orthopedic surgery, and to do some modifications to make the system more accurate in predicting postoperative complication rates.

**Methods:**

This is a retrospective clinical study involving 779 patients between April 1, 2009 and September 1, 2010. The postoperative complication rates were predicted by POSSUM, and then compared with the actual morbidity. Logistic regression was taken to improve the POSSUM equation.

**Results:**

In the 779 cases, the predicted morbidity was 27.2 % (212 cases) by POSSUM, while the actual morbidity is 8.3 % (65 cases).

**Conclusion:**

POSSUM excessively predicted the morbidity of patients undergoing orthopedic surgery, and it could be more accurate with appropriate modification. Of all risk factors, echocardiography ejection fraction showed a close relationship with postoperative complications.

## Introduction

Physical and operative severity score for the enumeration of mortality and morbidity (POSSUM) scoring system was first created by Copeland in 1991 [1]. The scoring system was widely used in general surgery as it could predict morbidity and mortality. Since POSSUM excessively predicted the postoperative morbidity and mortality, the modified version P-POSSUM was born [2]. V-POSSUM for vascular surgery, RAAA-POSSUM for abdominal aortic aneurysm repair, O-POSSUM for esophageal surgery, Cr-POSSUM for colorectal surgery, and a modified POSSUM scoring system for spinal surgery were also proposed [3–7]. However, POSSUM scoring system have several defects. First, the scoring system only could be used in patients undergoing surgery. Second, it may overestimate the postoperative mortality. Third, mortality more than 30 days could be not predicted.

With the development of medical science, pulmonary function test, echocardiography, ultrasound of lower extremity vascular, ultrasound of carotid, SPECT-CT, etc., were born. Compared to traditional inspection methods, the new medical equipment are more sensitive and accurate. To solve the above problems, we tried to make a new scoring system to predict morbidity and mortality more accurately for orthopedic patients by adding some items in POSSUM scoring system.

## Materials and methods

A total of 779 consecutive patients who had undergone orthopedic surgery between April 1, 2009 and September 1, 2010 were included retrospectively in this study. The study was approved by the ethics committee in our hospital. We included 334 men and 445 women ranging in age from 12 to 96 years with a mean of 61.0 years. Among them, there were 207 cases of the lumbar spine disease, 40 cases of cervical disease, 86 of joint disease, 255 of fracture, 15 of peripheral nerve entrapment, 51 of tumor, 88 of sports injuries, 3 of infectious diseases, and 34 of other diseases. For every included patient, a file was created. All the related data, including patient demographic data (medical record number, admission date, discharge date, date of surgery), admission/discharge diagnosis, surgeon, surgical approach, physiological scores and operative severity scores of POSSUM, other related indicators (preoperative comorbidity, serum albumin, serum creatinine, body mass index, time of hospitalization, ejection fraction, pulmonary one second volume, blood oxygen values), and mortality during period of hospitalization, morbidity occurred within 30 days after surgery, were recorded.

POSSUM scoring system consists of 12 physiological scores and 6 operative severity scores, as shown in Table 1. Equation for predicting morbidity was lnR1/(1-R1) = −7.04 + (0.13 × physiological score) + (0.16 × operative severity score) (R1 represents morbidity). Some new items were added in the analysis such as serum albumin, serum creatinine, body mass index, hospital stay, ejection fraction, pulmonary one second volume, and blood oxygen values. We analyzed the relationship between the new items and postoperative complications in patients. Logistic regression was taken to obtain a new equation for predicting morbidity.

**Table 1:** POSSUM Scoring System

	Score			
	1	2	4	8
Physiological scores				
Age (years)	≤60	61–70	≥71	
Cardiac signs	Normal	Cardiac drugs or steroid	Edema Warfarin	Raised jugular venous pressure
Resp signs	Normal	SOB exertion	SOB stairs	SOB rest
		Mild COPD	Mod COPD	Other changes
Systolic BP (mmHg)	110–130	131–170	≥171	≤89
		100–109	90–99	
Pulse (/min)	50–80	81–100	101–120	≥121
		40–49		≤39
Glasgow scores	15	12–14	9–11	≤8
Blood urea (mmol/l)	≤7.5	7.6–10	10.1–15	≥15.1
Blood Na^+^ (mmol/l)	≥136	131–135	126–130	≤125
Blood K^+^ (mmol/l)	3.5–5	3.2–3.4	2.9–3.1	≥6
		5.1–5.3	5.4–5.9	≤2.8
Hb (g/100 ml)	130–160	115–129	100–114	≥181
		161–170	171–180	≤99
White cell count (×10^9^/l)	4–10	10.1–20	≥20.1	
		3.1–3.9	≤3	
ECG	Normal		Atrial fibrillation (60–90)	Any other changes
Operative severity score				
Magnitude	Minor	Inter	Major	Major+
Number of operative variables within 30 days	1		2	≥3
Blood loss (ml)	≤100	101–500	501–999	≥1000
Contamination Abdominal cavity infection	None	Incised wound	Minor contamination	Gross contamination
Presence of malignancy	None	Primary	Node metastases	Distant metastases
Timing of operation	Elective		Emergency (<24 h)	Emergency (<6 h)

*SOB* shortness of breath, *COPD* chronic obstructive pulmonary disease

A database involving a total of 779 consecutive patients were established by Epidata 3.1. In order to predict postoperative morbidity or mortality from the lowest risk to the highest risk, 10 risk bands were divided. Each risk band contained the same number of subjects. Expected and observed complications or death were quantified in each band. Then the observed morbidity rate and the expected morbidity rate ratio were calculated (OE = observed complication rate/expected complication rate). SPSS 17.0 software (Chicago, IL, USA) was used to analyze the data. Missing values were defined as normal (score = 1). Physiologic and operative severity scores were described by mean ± standard deviation. Data was compared by the *χ*2 test. Multivariate analysis was performed using the logistic regression method. *P* <0.05 was considered statistically significant. The discriminatory power was assessed by the area under the curve (AUC). Values ranging from 0.5 to 0.7 showed a lower diagnostic accuracy, 0.7 to 0.9 represented a moderate diagnostic accuracy, and AUC > 0.9 was a higher diagnostic accuracy.

## Results

Within 30 days after surgery, a total of 82 complication cases happened in 65 patients, including 8 cases in myocardial infarction, heart failure, arrhythmia, and other cardiac complications, 19 cases in respiratory failure, pneumonia, and other respiratory complications, 14 cases in cerebral infarction, delirium, and other cerebrovascular complications, 16 cases in wound infection, seroma, infection with implant and bedsores, 8 cases in peptic ulcer, cholecystitis, pancreatitis, and other gastrointestinal complications, 11 cases in lower limb venous thrombosis, 6 cases in urinary tract infections, urinary retention, urinary tract complications. In addition, 2 patients died during the hospital stay.

The average physiologic score was 17.2 ± 4.36, and the average operative severity score was 10.4 ± 3.70, which predicted the probability of complication rate was 27.2 % (212 cases). However, the actual complication rate was 8.3 % (65 cases). Besides, there was a significant difference between the observed complication rate and the expected complication rate, (*χ*^2^ = 94.88, *P* < 0.001). In a word, POSSUM overpredicted the morbidity in orthopedic patients. The observed morbidity and the expected morbidity (OE ratio) were shown in Table 2. From Table 2, we know that POSSUM was not a sensitive predictor for the actual complication rates in orthopedic patients.

**Table 2:** Comparison of the observed value and the expected value

Expected rate %	Cases	The average expected rate (%)	The number of expected cases	The number of observed cases	OE ratio
0–10	163	7.67	12	2	0.17
10–20	199	14.6	29	9	0.31
20–30	125	24.2	30	8	0.27
30–40	103	35.0	36	15	0.42
40–50	84	44.0	37	8	0.22
50–60	48	54.6	26	8	0.31
60–70	31	65.0	20	8	0.40
70–80	13	74.5	10	5	0.50
80–90	9	83.0	7	2	0.29
90–100	4	94.0	4	0	0

For physiologic score (PSS) and operative severity score (OSS), preoperative comorbidity, serum albumin, serum creatinine, body mass index, time of hospitalization, ejection fraction, pulmonary one second volume, and blood oxygen values with the actual morbidity were related indicators. However, only echocardiographic ejection fraction have a significant correlation with actual morbidity (*P* <0.001). The ejection fraction was divided into 4 grades: >65 % = 1 score, 55–65 % = 2 score, 40–55 % = 4 score, <40 % = 8 score. Moreover, the 4 grades had a significant correlation with actual morbidity (*P* <0.001). Therefore, echocardiography ejection fraction had a significant correlation with the prognosis of orthopedic patients.

**Fig. 1:**
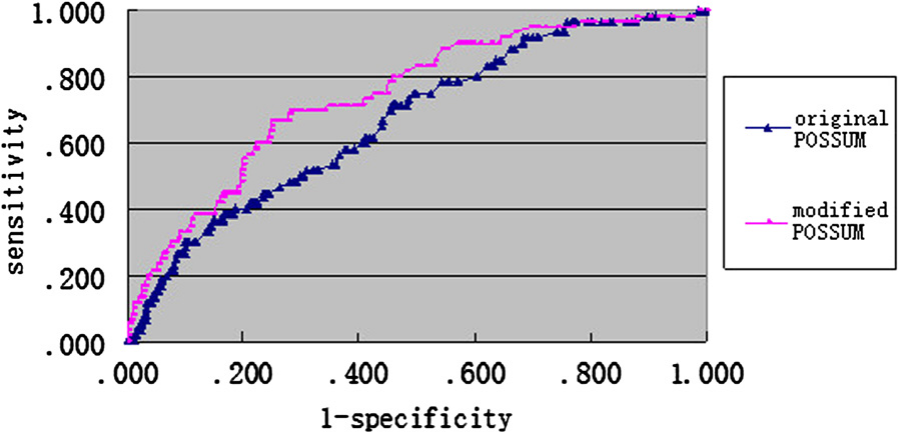
AUC of original equation and modified equation

Ejection fraction values, OSS and PSS were used as independent variables, then we got a modified equation to predict morbidity: ln (P/1-P) = −6.112 + 1.244 × echocardiographic ejection fraction score + 0.096 × PSS + 0.044 × OSS (*P* for morbidity). The AUC of modified equation was 0.746 and the AUC of original equation was 0.670 (Fig. 1), which indicated that both the two equations could predict postoperative morbidity, and that the modified equation was better.

In order to test the equation’s accuracy, another 84 patients with hip fracture in our institute were collected. The morbidity was 27.39 % (23 cases) for original equation and 15.07 % (13 cases) for modified equation. However, the actual complication cases were 15 for the 84 hip fracture patients. There was a significant difference between the observed morbidity and expected morbidity (*χ*^2^ = 94.88, *P* < 0.001). So, the modified equation had a better predictive ability than the original one, and it was closer to the actual complication rates.

## Discussion

Nowadays, it is still a difficult to predict postoperative mortality and morbidity and to make a decision whether or not some patient can withstand surgery. However, surgeons need an objective, comprehensive, and more accurate risk assessment system, which can be helpful in selecting the appropriate treatment for some patient. Moreover, the most important thing is that the system also can reduce the postoperative morbidity and mortality.

POSSUM scoring system is an evaluation system which originated in the UK and formed by statistical calculations of hundreds of thousands of patients data [1]. After that it was proved to be widely used in general surgery, cardiothoracic surgery, vascular surgery, orthopedic surgery, and other fields and can accurately predict morbidity and mortality [8–11]. However, POSSUM scoring system over predicted postoperative mortality in orthopedic patients [12]. Moreover, the current study also confirmed that POSSUM scoring system overpredicted the postoperative morbidity.

POSSUM scoring system was developed in 1991, and it has been widely approved. But during the passed 20 years, the population aging and disease has changed dramatically. With progress of medical science, there are large changes in the evaluation of the preoperative physiologic conditions, such as pulmonary function test, echocardiography, ultrasound of lower extremity vascular, ultrasound of carotid, SPECT-CT, etc. Compared to traditional inspection methods, the new medical equipment are more sensitive and accurate. As we know, the most significant finding of the present study was that echocardiography ejection fraction in patients had a significant correlation with postoperative morbidity. Thus, we added new items in the modified POSSUM scoring system.

There are several limitations of the current study: (1) it was a retrospective study, and all the data came from a district teaching hospital’s orthopedic ward. (2) The sample size of the current study was still not large enough to divide into groups by cardiac, respiratory, and other complications, which may have low power. Therefore, multi-disciplinary, multi-center, and large sample size studies are also needed to confirm the new scoring system.

In conclusion, POSSUM excessively predicted the morbidity of patients undergoing orthopedic surgery, and it could be more accurate with appropriate modification. However, based on the limits of the current study, large sample, multi-center studies are still needed.
